# Local health department engagement with workplaces during the COVID-19 pandemic—Examining barriers of and facilitators to outbreak investigation and mitigation

**DOI:** 10.3389/fpubh.2023.1116872

**Published:** 2023-03-17

**Authors:** Tessa Bonney, Michael P. Grant

**Affiliations:** ^1^Division of Environmental and Occupational Health Sciences, School of Public Health, University of Illinois Chicago, Chicago, IL, United States; ^2^Division of Field Studies and Engineering, National Institute for Occupational Safety and Health, Centers for Disease Control and Prevention, Cincinnati, OH, United States

**Keywords:** COVID-19, occupational exposure, public health practice, prevention, workplace, SARS-CoV-2

## Abstract

**Objectives:**

To document local health department (LHD) COVID-19 prevention or mitigation activities at workplaces in the United States and identify facilitators for and barriers to these efforts.

**Methods:**

We conducted a web-based, cross-sectional national probability survey of United States LHDs (*n* = 181 unweighted; *n* = 2,284 weighted) from January to March 2022, collecting information about worker complaints, surveillance, investigations, relationships and interactions with employers/businesses, and LHD capacity.

**Results:**

Overall, 94% LHD respondents reported investigating workplace-linked COVID-19 cases; however, 47% reported insufficient capacity to effectively receive, investigate and respond to COVID-19-related workplace safety complaints. Prior relationships with jurisdiction employers and LHD personnel with formal occupational health and safety (OHS) training were predictors of proactive outreach to prevent COVID-19 spread in workplaces (*p* < 0.01 and *p* < 0.001). LHD size predicted OHS personnel and sufficient financial resources to support workplace investigation and mitigation activities (*p* < 0.001).

**Conclusions:**

Differences in LHD capacity to effectively respond to communicable disease spread in workplaces may exacerbate health disparities, especially between rural and urban settings. Improving LHD OHS capacity, especially in smaller jurisdictions, could facilitate effective prevention and mitigation of workplace communicable disease spread.

## 1. Introduction

The COVID-19 pandemic caused unprecedented communicable disease exposure in workplaces, with prolonged workplace outbreaks emerging across the United States especially in certain “essential” industries (e.g., manufacturing and wholesale trade) that disproportionately employ low-wage workers of color (1, 2). Factors such as indoor, close-proximity work, coupled with the concentration of jobs with low wages, no healthcare benefits or paid sick leave in densely populated areas with higher rates of multi-generational, crowded housing increased the risks of exposure to SARS-CoV-2, the virus that causes COVID-19, among these workers (3). While human contact patterns outside of households changed drastically early in the pandemic, contact rates at many workplaces remained elevated, especially in “essential” industries (4). Studies that examined associations between household members of workers in at-risk occupations and household cases (5) or COVID-19-related hospital admissions (6) highlight the risks or workplace to home transmission of COVID-19.

Workplace outbreaks of COVID-19 presented a novel challenge to employers and public health departments, whereby personnel at most workplaces had little experience controlling airborne infectious disease exposures and health department personnel had limited prior interactions with non-healthcare or non-congregate workplaces to prevent and mitigate such exposures. Case studies from various industries highlight the challenges that workplaces encountered when attempting to reduce exposures, such as inadequate ventilation and inappropriate administrative controls in nursing homes (7) and lack of compliance with masking or available ventilation controls in schools, along with barriers to installation of systems to reduce SARS-CoV-2 transmission (8, 9). In order to identify persons with COVID-19 with exposures linked to workplaces and prioritize workplaces for investigation and supports, the United States Centers for Disease Control and Prevention (CDC) recommended that health departments employ a prioritization scheme for identifying persons with COVID-19 and their contacts at non-healthcare workplaces (10, 11). While at least one health department described the development and implementation of such an approach (12), there is an absence of literature documenting other health departments' approaches to identifying persons with COVID-19 linked to their workplaces or the activities that departments undertook to prevent and mitigate COVID-19 in workplaces in their jurisdictions.

Local health departments (LHDs) were tasked with investigating and intervening in outbreaks that they did identify while simultaneously maintaining their pre-pandemic duties, including monitoring community health status and providing essential services to solve wide-ranging health problems in their catchment areas, for which LHD capacity was already limited (13, 14). Reports from the first year of the COVID-19 pandemic highlighted the disruptions in the continuity of essential health services globally (15, 16), and the reduction of or disruption to preventive health services resulted in documented reductions in cancer screenings (17), decreases in and delays in appropriate care for individuals with chronic diseases (18), and excess mortality from non-COVID-19 diseases (19). Especially in rural communities, LHDs serve as key providers of, and in some cases the only providers of, clinical services (20).

There is little literature on public health engagement with workplaces to prevent and mitigate workplace outbreaks of communicable diseases in the modern era. Communicable disease outbreaks and clusters in workplaces described in the pre-COVID-19 literature were limited, and many were industry-specific, readily contained, and easily traced and mitigated by local public health authorities (21). The COVID-19 pandemic thus serves as an opportunity to understand LHD actions in response to widespread transmission of SARS-CoV-2 in workplace settings, as well as potential gaps in LHD readiness and capacity to engage in workplace-based mitigation or prevention efforts. To fill this gap, this study sought to (A) document LHD actions related to prevention or mitigation of COVID-19 cases and exposures at workplaces and (B) examine facilitators for and barriers to COVID-19 workplace outbreak investigation and mitigation efforts completed by LHDs.

## 2. Methods

We conducted a web-based, cross-sectional national probability survey of United States (U.S.) LHDs from January to March 2022. Survey development began mid-2021 and included two rounds of content-focused review and pre-testing with subject matter experts from the National Institute for Occupational Safety and Health (NIOSH) and from the Occupational Health Sub-committee of the Council of State and Territorial Epidemiologists. We employed a stratified sampling design to draw a random sample of 1,214 LHDs from the National Association of County and City Health Officials (NACCHO) Directory of LHDs, a comprehensive list of U.S. LHDs, with strata defined by U.S. Census geographical region (Northeast, South, Midwest, West) and by population size served (three categories based on 2019 U.S. Census population estimates: < 50,000, 50,000–499,999, ≥500,000) (22). We sampled LHDs with email contacts listed in the NACCHO Directory, and we sampled at the district or regional level in states with public health decision making directed partially or completely by state-level decision makers (e.g., centralized, shared, or mixed governance structures) (23). Given considerable COVID-19-related strain on LHDs, we anticipated a low response rate across strata and thus oversampled strata with fewer LHDs to maximize statistical power in the final sample.

The survey questionnaire included 44 items across the following domains: (i) COVID-19-related complaints in workplaces; (ii) surveillance of occupational exposures and known positive cases by industry and occupation; (iii) workplace-based investigations of outbreaks; (iv) LHD relationships and interactions with employers or business establishments; and (v) LHD capacity and organizational characteristics. Survey items were primarily closed-ended multiple-choice or multiple-selections, with a small number of open-ended items. The survey was administered *via* Qualtrics software (Version 2022, Provo, UT).

To better characterize LHD respondents in our analyses, we added a governance structure variable (i.e., decentralized vs. centralized, shared, or mixed). To produce nationally representative estimates, we generated sampling weights for each stratum, accounting for oversampling of and differential response rates by strata. We calculated descriptive statistics for LHD responses to capacity measures and measures of LHD engagement with workers and businesses in the aggregate, representing categorical LHD characteristics and survey responses as weighted proportions and 95% confidence intervals (CIs). The survey resulted in several capacity metrics (e.g., personnel, relationships with workplaces) and worker or business engagement activities. We constructed simple logistic regression models to assess: (1) which LHD characteristics were associated with select capacity metrics and (2) which capacity metrics were associated with the following activities: (a) collected industry or occupation information at testing or through contact tracing; (b) received requests to evaluate engineering controls, such as ventilation systems; (c) conducted in-person investigations of workplaces in response to worker complaints; and (d) enlisted the support of enforcement agencies, such as state or federal Occupational Safety and Health Administration (OSHA) offices. We selected these activities based on their likelihood to be most impactful in reducing risk of exposure to SARS-CoV-2 in the workplace (e.g., identifying and removing persons with COVID-19 from the workplace, employing engineering controls to remove viral particles from the air, enforcing COVID-19-related emergency temporary standards where applicable). We used SPSS Statistical Software version 26.0 (IBM, 2019) for all analyses. This activity was reviewed by CDC and was conducted consistent with applicable federal law and CDC policy [see e.g., 45 C.F.R. part 46.102(l)(2), 21 C.F.R. part 56; 42U.S.C. §241(d); 5 U.S.C. §552a; 44 U.S.C. §3501 et seq.].

## 3. Results

One-hundred eighty of 1,214 sampled LHDs (15%) responded to the survey. Response rate was highest among LHDs from the West region (11%), followed by Midwest and Northeast regions (8%, respectively), and lowest in the South region (6%). LHDs with more populous jurisdictions had the highest response rate (17% for populations ≥500,000), followed by medium-sized jurisdictions (10% for populations 50,000–499,999) and smaller jurisdictions (6% for populations < 50,000). Respondents most frequently represented non-metropolitan areas (*n* = 99), jurisdictions with 50,000–499,999 residents (*n* = 78), the Midwest region (*n* = 67), and decentralized governance structures (*n* = 152; see Table 1).

**Table 1:** Characteristics of participating LHDs (*n* = 180 unweighted; *n* = 2,284 weighted).

**Characteristics**	**No. LHDs (unweighted)**	**% LHDs (weighted)**
**Jurisdiction type** ^*^		
Non-metropolitan	99	67.6
Metropolitan	81	32.4
**Size of population served**		
< 50,000	73	59.5
50,000–499,999	78	33.5
≥500,000	29	6.9
**U.S. Census geographical region**		
Northeast	47	25.0
South	30	22.6
Midwest	67	38.3
West	36	14.1
**State and LHD governance**		
Centralized, shared, or mixed	28	16.1
Decentralized	152	83.9

Data are weighted to account for oversampling and differential response rates by strata.

^*^Metropolitan classification defined as jurisdictional area within a metropolitan statistical area as defined by the U.S. Office of Management and Budget (OMB). Non-metropolitan classification assigned to jurisdictional areas that do not include or are not within a metropolitan statistical area.

### 3.1. LHD capacity

Approximately half of LHDs reported that they have the capacity to receive, investigate, and respond to workplace safety complaints (52.5%; 95% CI, 50.4%−54.5%), and fewer than half reported that they have the financial resources necessary to adequately support workplace investigation and mitigation activities (41.8%; 95% CI, 39.8%−43.9%; see Table 2). Fewer than half of all LHDs reported personnel on staff with formal occupational health and safety (OHS) training and expertise, such as training in exposure assessment, industrial hygiene, occupational safety, occupational medicine, or occupational nursing (40.9%; 95% CI, 38.9%−43.0%). The majority of LHDs reported reassigning personnel (70.4%; 95% CI, 68.4%−72.3%) or hiring new personnel (71.6%; 95% CI, 69.7%−73.5%) to assist with workplace investigation and mitigation activities during the COVID-19 pandemic (defined as the period beginning March 2020 and on-going at time of data collection); however, less than a third reported having a dedicated taskforce for workplace investigation and mitigation activities.

**Table 2:** Capacity metrics reported by LHDs (*n* = 180 unweighted; *n* = 2,284 weighted).

**Capacity measure**	**% (95% CI)**
**Personnel**	
Personnel with formal OHS^I^ training and expertise	40.9 (38.9–43.0)
Dedicated taskforce for workplace investigation and mitigation	31.7 (29.7–33.7)
Reassigned personnel for workplace investigation and mitigation	70.4 (68.4–72.3)
Hired new personnel for workplace investigation and mitigation	71.6 (69.7–73.5)
**Relationships**	
Routine engagement with employers/business establishments pre-pandemic	69.4 (67.5–71.3)
Positive relationships with local authorities	95.3 (94.4–96.2)
**Overall**	
Capacity to receive, investigate, and respond to workplace safety complaints	52.5 (50.4–54.5)
Sufficient financial resources to support workplace investigation and mitigation	41.8 (39.8–43.9)

Data are weighted to account for oversampling and differential response rates by strata. Percent and 95% confidence intervals reported in table represent weighted data.

^I^Occupational health and safety training and expertise (e.g., exposure assessment, industrial hygiene, occupational safety, occupational medicine, or occupational nursing).

### 3.2. Engagement with workers, employers, and business establishments

Approximately half of all LHDs reported consistent collection of occupation or industry information from individuals through LHD-administered contact tracing programs (53.4%; 95% CI, 51.4%−55.5%), and about one third reported collection of occupation or industry information at LHD-administered testing sites (32.4%; 95% CI, 30.4%−34.3%; see Table 3). Fewer than half of all jurisdictions required employers to report known numbers of persons with COVID-19 to the LHD (46.6%; 95% CI, 44.6%−48.7%). While over 90% of LHDs reported investigating workplace-linked COVID-19 outbreaks, fewer than half of those LHDs reported that these investigations were conducted in-person at least some of the time (41.6%; 95% CI, 39.5%−43.7%). LHDs reported lack of or inconsistent enforcement of masking mandates and social distancing as the most frequent complaints from workers, while only 44.1% of LHDs (95% CI, 41.8%−46.5%) reported receiving complaints about appropriate engineering controls, such as venti lation, or about training specifically focused on COVID-19 prevention in the workplace. The majority of LHDs that received complaints from workers reported direct, remote engagement with employers to resolve complaints (86.0%; 95% CI, 84.3%−87.6%). Fewer than half of LHDs reported enlisting supports from outside agencies, such as enforcement agencies or non-enforcement public health agencies, to help resolve complaints (42.8%; 95% CI, 40.5%−45.2%). Nearly all LHDs also reported receiving requests for support from employers (96.1%; 95% CI, 95.2%−96.8%), with most requests focused on specific guidance for minimizing spread of COVID-19 in workplaces. In addition to requests for written information and signage, LHDs reported receiving requests to support on-site vaccination efforts (68.1%; 95% CI, 66.0%−70.1%), to connect workers with vaccinations off-site (73.7%; 95% CI, 71.8%−75.6%), and to support contact tracing (57.9%; 95% CI, 55.8%−60.1%) and testing efforts (52.4%; 95% CI, 50.2%−54.5%).

**Table 3:** Proportion of LHDs reporting engagement with workers and/or employment establishments (*n* = 180 unweighted; *n* = 2,284 weighted).

**Activity**	**% (95% CI)**
**Surveillance**	
Occupation/industry consistently collected at LHD-administered contact tracing	53.4 (51.4–55.5)
Occupation/industry consistently collected at LHD-administered testing sites	32.4 (30.4–34.3)
Employers in jurisdiction required to report known positive cases to LHD	46.6 (44.6–48.7)
**Investigations**	
LHD has investigated COVID-19 cases linked to workplaces	94.7 (93.7–95.6)
**Location of investigations**	
In-person, at the workplace only	0.8 (0.5–1.3)
Remotely (e.g., telephone, video call, email) only	57.6 (55.5–59.7)
Both in-person and remotely	41.6 (39.5–43.7)
**Complaints from workers**	
LHD received complaints from workers	76.9 (75.2–78.7)
**Type(s) of complaints**	
Lack of or inconsistent enforcement of masking mandates	92.7 (91.4–93.9)
Lack of or inconsistent enforcement of social distancing at the workplace	86.0 (84.3–87.6)
Lack of cleaning and sanitizing procedures at the workplace	71.1 (68.9–73.2)
Failure to require and/or provide appropriate PPE^a^ for workers	57.6 (55.3–60.0)
Absence of employee pre-work screenings for COVID-19	47.6 (45.3–50.0)
Lack of appropriate engineering controls (e.g., ventilation in enclosed spaces)	44.1 (41.8–46.5)
Lack of or inadequate training about COVID-19 prevention in the workplace	37.7 (35.5–40.1)
**Action(s) to address complaints**	
Direct outreach to employers/establishments to resolve complaints remotely	86.0 (84.3–87.6)
Consulted with your state health department	58.8 (56.5–61.2)
Conducted in-person investigations in response to specific worker complaints	53.2 (50.8–55.5)
Enlisted the assistance of other enforcement authorities^b^	42.8 (40.5–45.2)
Referred complaints to another agency or authority	27.9 (25.8–30.1)
Consulted with Centers for Disease Control and Prevention (CDC) or NIOSH	10.1 (8.7–11.6)
Engaged with worker unions/advocacy groups to resolve complaints	7.2 (6.0–8.5)
**Supports requested by employers**	
LHD received request for support from employers/business establishments	96.1 (95.2–96.8)
Specific guidance or recommendations for preventing spread of COVID-19 in the employers' establishment(s)^c^	95.6 (94.6–96.4)
Written information about best-practices for preventing spread of COVID-19	82.0 (80.4–83.7)
Connecting employers and employees to off-site vaccination sites	73.7 (71.8–75.6)
Signage about COVID-19 and how to minimize its spread^d^	71.2 (69.1–73.1)
On-site vaccination support	68.1 (66.0–70.1)
Assistance with business establishment's contact tracing efforts	57.9 (55.8–60.1)
Assistance with on-site employee testing for COVID-19	52.4 (50.2–54.5)

Data are weighted to account for oversampling and differential response rates by strata. Percent and 95% confidence intervals reported in table represent weighted data.

^a^Personal protective equipment.

^b^E.g., federal or state Occupational Safety and Health Administration (OSHA) or other state or local health authorities.

^c^E.g., establishing cleaning protocols, assessing ventilation, making PPE recommendations, determining indoor capacity.

^d^E.g., signage about masking, social distancing, vaccination.

### 3.3. LHD characteristics as predictors of LHD capacity

There were several notable differences in reported LHD capacity by jurisdiction characteristic (see Table 4). Metropolitan-serving LHDs were more than twice as likely than those serving non-metropolitan jurisdictions to employ personnel with formal OHS training and expertise (OR = 2.7; 95% CI, 2.2–3.2). LHDs from increasingly populous jurisdictions were also significantly more likely to employ personnel with OHS training and expertise (population 50,000–499,999: OR = 1.9; 95% CI, 1.6–2.4; population ≥500,000: OR = 7.1; 95% CI, 5.0–10.0). The LHDs serving jurisdictions with at least 500,000 residents were twice as likely as those serving < 50,000 residents to have a dedicated taskforce for workplace investigation and mitigation activities; they were more than twice as likely to report having the capacity to receive, investigate, and respond to workplace safety complaints (OR = 2.4; 95% CI, 1.7–3.4); and they were twice as likely to report having sufficient financial resources to support workplace investigation and mitigation activities (OR = 2.1; 95% CI, 1.5–2.9). Jurisdictions in the South, Midwest, and West regions were all more likely to report having a dedicated taskforce for workplace investigation and mitigation activities (ORs = 6.2, 3.1, and 4.7) and having sufficient financial resources to support those activities (ORs = 2.9, 2.6, and 3.9) than jurisdictions in the Northeast. Jurisdictions in the Midwest and West were also more likely to report having the capacity to receive, investigate, and respond to workplace safety complaints than those in the Northeast (ORs = 2.0 and 1.9). Decentralized LHDs were less likely to report having a dedicated taskforce for workplace investigation and mitigation activities than those with other governance structures (OR = 0.7, 95% CI, 0.5–0.8) and were more likely to report having the capacity to handle complaints.

**Table 4:** Associations of LHD characteristics with select capacity measures (*n* = 180 unweighted; *n* = 2,284 weighted), OR (95% CI).

**Characteristics**	**Personnel with formal OHS training and expertise**	**Dedicated taskforce for workplace investigation and mitigation**	**Capacity to receive, investigate and respond to workplace safety complaints**	**Sufficient financial resources to support workplace investigation and mitigation**
**Jurisdiction type** ^*^				
Non-metropolitan	Ref.	Ref.	Ref.	Ref.
Metropolitan	2.7 (2.2–3.2)^a^	1.1 (0.9–1.4)	1.0 (0.8–1.2)	1.2 (1.0–1.4)
**Size of population served**				
< 50,000	Ref.	Ref.	Ref.	Ref.
50,000–499,999	1.9 (1.6–2.4)^a^	0.8 (0.7–1.0)	1.2 (1.0–1.5)	1.0 (0.9–1.3)
≥500,000	7.1 (5.0–10.0)^a^	2.2 (1.6–3.1)^a^	2.4 (1.7–3.4)^a^	2.1 (1.5–2.9)^a^
**U.S. Census geographical region**				
Northeast	Ref.	Ref.	Ref.	Ref.
South	1.2 (0.9–1.5)	6.2 (4.6–8.5)^a^	1.0 (0.8–1.3)	2.9 (2.2–3.8)^a^
Midwest	0.9 (0.7–1.1)	3.1 (2.3–4.2)^a^	2.0 (1.6–2.5)^a^	2.6 (2.0–3.2)^a^
West	1.1 (0.8–1.5)	4.7 (3.3–6.6)^a^	1.9 (1.4–2.5)^a^	3.9 (2.9–5.2)^a^
**State and LHD governance**				
Centralized, shared, or mixed	Ref.	Ref.	Ref.	Ref.
Decentralized	1.3 (1.0–1.6)	0.7 (0.5–0.8)^b^	2.2 (1.7–2.8)^a^	1.0 (0.8–1.3)

Data are weighted to account for oversampling and differential response rates by strata. Odds ratios and 95% confidence intervals reported in table represent weighted data.

^*^Metropolitan classification defined as jurisdictional area within a metropolitan statistical area as defined by the U.S. Office of Management and Budget (OMB). Non-metropolitan classification assigned to jurisdictional areas that do not include or are not within a metropolitan statistical area.

^a^p < 0.001.

^b^p = 0.0001.

### 3.4. LHD capacity metrics as predictors of engagement with workers, employers, and business establishments

LHD capacity was significantly associated with several notable workplace-facing activities, shown in Table 5. LHDs that reported personnel with formal OHS training and expertise were significantly more likely to collect industry or occupation when contact tracing or conducting testing (OR = 1.8; 95% CI, 1.5–2.1), to report receiving requests to evaluate engineering controls (OR = 2.0; 95% CI, 1.6–2.4), and to conduct in-person investigations in response to complaints (OR = 2.4; 95% CI, 1.9–2.9) than those that did not. LHDs with a dedicated taskforce for workplace investigation and mitigation activities were also more likely to collect industry or occupation (OR = 1.6; 95% CI, 1.4–2.0) and to report requests to evaluate engineering controls (OR = 2.0; 95% CI, 1.7–2.5). LHDs that reassigned personnel for workplace investigation and mitigation activities were more likely to engage in all workplace-facing activities reported in Table 5 than LHDs that did not reassign personnel (ORs ranging from 1.3 to 1.9). LHDs that hired new personnel for these activities were more than twice as likely as those that did not to collect industry or occupation (OR = 2.3; 95% CI, 1.9–2.8) and to conduct in-person investigations of complaints (OR = 2.5; 95% CI, 2.0–3.1), and they were less likely to enlist support of enforcement agencies (OR = 0.7; 95% CI, 0.6–0.9). LHDs that reported routine engagement with employers prior to the pandemic were more likely to collect industry or occupation (OR = 2.1; 95% CI, 1.7–2.5) and were more likely to report requests to evaluate engineering controls (OR = 1.5; 95% CI, 1.2–1.9), as were LHDs that reported positive relationships with local authorities (ORs = 1.8; 95% CI, 1.1–2.7, and 3.2; 95% CI, 2.0–5.7). The latter group was also more likely to report conducting in-person investigations relative to LHDs that did not report positive relationships with local authorities (OR = 2.5; 95% CI, 1.6–3.9). LHDs that reported an overall capacity to receive, investigate, and respond to workplace safety complaints were nearly twice as likely to collect industry or occupation (OR = 1.9; 95% CI, 1.6–2.2) and to conduct in-person investigations at workplaces (OR = 1.9; 95% CI, 1.5–2.3). Those that reported having sufficient financial resources to support investigation and mitigation activities were also more likely to collect industry or occupation (OR = 2.5; 95% CI, 2.1–3.0) and were more likely to report requests for evaluation of engineering controls (OR = 1.6; 95% CI, 1.3–1.9).

**Table 5:** Associations of LHD capacity with select workplace-facing activities (*n* = 180 unweighted; *n* = 2,284 weighted), OR (95% CI).

**Capacity measure**	**Industry/occupation collected as part of surveillance activities^ᵻ^**	**Reported receiving requests to evaluate engineering controls^ᵻᵻ^**	**Conducted in-person investigations in response to worker complaints**	**Enlisted supports of enforcement agencies^ᵻᵻᵻ^**
**Personnel**				
Personnel with formal OHS training and expertise	1.8 (1.5–2.1)^a^	2.0 (1.6–2.4)^a^	2.4 (1.9–2.9)^a^	0.8 (0.7–1.0)
Dedicated taskforce for workplace investigation and mitigation	1.6 (1.4–2.0)^a^	2.0 (1.7–2.5)^a^	0.9 (0.7–1.1)	1.0 (0.8–1.3)
Reassigned personnel for workplace investigation and mitigation	1.5 (1.2–1.8)^a^	1.3 (1.1–1.7)^b^	1.8 (1.5–2.3)^a^	1.9 (1.5–2.3)^a^
Hired new personnel for workplace investigation and mitigation	2.3 (1.9–2.8)^a^	1.0 (0.8–1.2)	2.5 (2.0–3.1)^a^	0.7 (0.6–0.9)^c^
**Relationships**				
Routine engagement with employers pre-pandemic	2.1 (1.7–2.5)^a^	1.5 (1.2–1.9)^a^	1.2 (1.0–1.5)	0.8 (0.7–1.0)
Positive relationships with local authorities (e.g., sheriff, police)	1.8 (1.1–2.7)^d^	3.2 (2.0–5.7)^a^	2.5 (1.6–3.9)^a^	0.9 (0.6–1.4)
**Overall**				
Capacity to receive, investigate and respond to workplace safety complaints	1.9 (1.6–2.2)^a^	1.1 (0.9–1.3)	1.9 (1.5–2.3)^a^	0.8 (0.7–1.0)
Sufficient financial resources to support workplace investigation and mitigation	2.5 (2.1–3.0)^a^	1.6 (1.3–1.9)^a^	0.8 (0.7–1.0)	0.8 (0.7–1.0)

Data are weighted to account for oversampling and differential response rates by strata. Percent and 95% confidence intervals reported in table represent weighted data.

^ᵻ^Industry and/or occupation consistently collected through LHD-administered contact tracing and/or testing sites.

^ᵻᵻ^Engineering controls including appropriate ventilation.

^ᵻᵻᵻ^Enforcement agencies including federal or state Occupational Safety and Health Administration (OSHA) or other state or local health authorities.

^a^p < 0.001.

^b^p = 0.004.

^c^p = 0.001.

^d^p = 0.01.

## 4. Discussion

Our study findings highlight the widespread engagement of LHDs with workplaces in the U.S. during the COVID-19 pandemic. While nearly all LHDs reported engagement with employers, business establishments, or workers in some capacity, we identified compelling differences in LHD capacity to surveil and intervene to prevent workplace spread of COVID-19 by jurisdiction type, size of population served, region, and LHD governance. We also found significant associations between these measures of capacity and select workplace-facing activities likely to be most effective in preventing or reducing exposure to SARS-CoV-2 (e.g., identifying known persons with COVID-19 and those exposed *via* testing and contact tracing; conducting thorough, in-person assessments of controls, especially engineering controls such as ventilation systems; enforcing health and safety regulations).

Despite ample evidence of occupation-based spread of COVID-19 early in the pandemic, nearly half of all LHDs did not report routine collection of industry or occupation for contact tracing purposes and less than a third reported collecting such information at LHD-administered testing sites. These statistics, coupled with the fact that employers were required to report known positive tests to fewer than half of all LHDs, highlight a notable missed opportunity to identify and potentially prevent workplace-based spread of COVID-19 across the U.S. by engaging with employers to reduce exposure risks in their establishments. When LHDs did identify COVID-19 cases linked to workplaces, the majority of LHDs reported conducting investigations of establishments remotely, potentially limiting their abilities to appropriately evaluate existing controls or to identify opportunities for more effective intervention to further reduce exposure risks in the physical workplace environment. In the future, collection of occupation and industry at testing and through contact tracing could help LHDs focus their investigative and mitigative efforts to ensure that workers at highest risk of infection are identified and better protected in their workplaces.

Compared with LHDs serving small (< 50,000) or medium (50,000–499,999) populations, LHDs serving large populations (≥500,000) reported an overall higher capacity to address the threat of COVID-19 in workplaces within their jurisdictions. LHDs in areas serving larger populations are likely to have a larger public health workforce (24), allowing them to more readily restructure and create a dedicated taskforce for workplace-facing activities. Larger LHDs may also be able to differentiate internal departments and attract more specialized personnel, such as individuals with formal OHS training. Employing personnel with OHS training and expertise was an important predictor of effective workplace-facing activities to prevent and reduce exposures, including collecting industry and occupation in surveillance activities, evaluating engineering controls, and conducting in-person investigations. Notably, LHDs that reported having OHS-trained personnel were less likely to report engagement with enforcement agencies, perhaps because these individuals are likely to be more familiar with regulatory standards and how to apply them in a workplace, or because these individuals may have the expertise required to operationalize CDC and state-level guidance. OHS training and expertise may be necessary to fulfill public health's core mission of preventing and controlling disease, specifically in a workplace context when airborne contaminants are of concern. Unlike the general public health workforce, those with OHS training and expertise are specifically trained to identify and evaluate hazards in the confines of a workplace and to select the most appropriate and effective available controls to address those hazards.

LHDs that reported routine engagement with employers prior to the pandemic and those that reported positive relationships with local authorities were also more likely to undertake high-impact workplace-facing activities, similar to our findings regarding employment of personnel with formal OHS training or expertise and having the agility to reconfigure their workforce. This finding aligns with strategies outlined in the Public Health 3.0 framework, specifically focused on the development and utilization of collaborative, strategic partnerships to effectively monitor and respond to drivers of population health (25). While robust relationships between these stakeholders was identified as a facilitator of LHD-workplace engagement, overall capacity to engage with workplaces and to fund such activities attenuate these findings. Only half of all LHDs reported overall capacity to receive, investigate, and respond to workplace safety complaints, and fewer than half reported sufficient financial resources to support efforts related to investigation and mitigation of COVID-19 in workplaces. Notably, workforce and funding needs of LHDs were not evenly distributed prior to the pandemic (26, 27), and this may have contributed to disparities in factors that facilitated workplace-focused prevention and mitigation efforts for COVID-19, such as having existing, collaborative relationships with business establishments from prior health assessment and planning activities.

LHDs in the Northeast were least likely to report sufficient financial resources, and LHDs in the Northeast and South were less likely to report overall capacity to receive, investigate, and respond to workplace safety complaints than LHDs in the Midwest and West. LHDs in states with decentralized governance structures were also more likely to report having an overall capacity to engage in these efforts. While perception of lack of capacity and resources is widespread, there may be regional-specific differences in demands (e.g., requests for support from employers, complaints from workers), LHD resources (e.g., funding, ability to attract and retain the public health workforce), or LHD decision latitude (e.g., enforcement authority, ability to enter workplaces). Centralized governance structures may reduce a LHD's ability to rapidly shift or reallocate resources to shore up capacity in these types of initiatives compared to LHDs where fiscal authority is at the local level.

While the threat of COVID-19 spread in workplaces galvanized the public health workforce to respond in a myriad of ways, it is evident that limitations in capacity constricted LHD's efforts. The chronic underfunding of the public health workforce and its impact on LHD readiness to respond to the COVID-19 pandemic is well-documented (28, 29), as are challenges related to prior cuts to and continuing decline of the public health workforce (14). More recently, researchers have highlighted the threat of further erosion of the public health workforce as burnout becomes more prevalent (30) and violence directed at public health officials, exacerbated by the continued politicization of the public health response to the COVID-19 pandemic, becomes increasingly common (31). It is thus important that we contextualize this study's findings in the realities facing public health, and that we recognize that the capacity limitations identified in this paper may not be easily remedied without increased financial resources for LHDs across the U.S. Further, and in the spirit of Public Health 3.0, there could be an examination of how the larger public health system, including enforcement and non-enforcement public health agencies and for-profit OHS firms that employ individuals with formal OHS training, might collaborate with LHDs to identify, evaluate, anticipate, and control biological hazards in workplaces.

This study is subject to several limitations. First, this study was designed to generate preliminary prevalence data about LHD interactions with workplaces during the pandemic and was not intended to provide an in-depth exploration nor evaluation of factors that facilitated or hindered LHD efforts related to the investigation and mitigation of COVID-19 spread in workplaces. Further, this study was not designed to examine LHD engagement with workplaces in specific communities or those with worker sociodemographic characteristics that were associated with higher rates of COVID-19 infection. The results presented here provide a foundation for more extensive, qualitative inquiry around these factors in follow-up studies. Additionally, the response rate in this study was fairly low (15%). The low response rate is not unexpected given the timing of survey deployment (recruitment began as the initial Omicron variant peaked in early 2022) and the resultant strain on LHDs across the country. Our methods of identifying and contacting the appropriate respondent for this survey were also imperfect and may have limited the survey response rate: contact information for LHD administrators was not always up to date and administrators may not have been the appropriate respondent at all LHDs, especially those serving larger jurisdictions. Despite these limitations, responses from LHDs provide initial insight into actions related to workplace prevention and mitigation of COVID-19 and highlight key facilitators of and barriers to these activities during the pandemic.

The findings and conclusions in this report are those of the authors and do not necessarily represent the official position of the National Institute for Occupational Safety and Health, Centers for Disease Control and Prevention.

## Data availability statement

The raw data supporting the conclusions of this article will be made available by the authors, without undue reservation.

## Ethics statement

The studies involving human participants were reviewed and approved by University of Illinois Chicago Institutional Review Board. The patients/participants provided their written informed consent to participate in this study.

## Author contributions

TB: conceptualization, methodology, formal analysis, investigation, and writing—original draft. MG: conceptualization and writing—review and editing. Both authors contributed to the article and approved the submitted version.
